# Book Reviews

**Published:** 1873-04-15

**Authors:** 


### Book Reviews.

The Science and Art of Surgery. By John Eric Erichson, Senior Surgeon to University College Hospital, London. New edition, revised and enlarged by the author; 700 engravings on wood. Philadelphia: H. C. Lea, Philadelphia.

The present edition comprises a work of 2,000 pages, and is in two volumes, the first embracing first principles and surgical injuries, and the second surgical diseases. This book has been long and favorably known to the profession, and it is, therefore, unnecessary to give a detailed account of the subjects treated. The present edition places the work fully abreast of the times in all the improvements of modern surgery. Many chapters are re-written and re-arranged, and much useful matter has been added. The chapter on inflammation and its results is changed to suit the advanced ideas of pathology; and the antiseptic treatment of wounds is fully given. An account of the transplantation of cuticle is also added. Diseases of the jaws are also more fully treated, and one or two new and useful illustrations of the methods of excision are given. This work has always been held in high estimation in the past, and the present edition fully entitles it to the confidence and support of the profession as a work of reference.

We acknowledge receipt of the following new works through Messrs. Jansen, McClurg & Co., 117 and 119 State st., Chicago:

A Manual of Chemical Analysis, as Applied to Examination of Medical Chemicals, a Guide for the Determination of their Identity and Quality, and for the Detection of Impurities and Adulterations. By Frederic Hoffman, P. H. D. New York: D. Appleton & Co.

Illustrations of the Influence of the Mind upon the Body in Health and Disease; designed to Elucidate the action of the Imagination. By Daniel H. Tuke, M.D., M.R.C.P. 8vo., 400 pp. Price $3.25. Philadelphia: H. C. Lea, publisher.

The following, we have received through Messrs. W. B. Keen, Cook & Co., 113 and 115 State st., Chicago:

Diseases of the Urinary Organs, including Stricture of the Urethra, Affections of the Prostates, and Stone in the Bladder. By John W. S. Gurley, M.D., with 103 engravings on wood. 8vo., 360 pp. New York: Wm. Wood & Co.

A Handbook of Post mortem Examinations, and of Morbid Anatomy. By Francis Delafield, M.D. 8vo., 368 pp. New York: Wm. Wood & Co.

